# COVID-19: A Recommendation to Examine the Effect of Mouthrinses with β-Cyclodextrin Combined with Citrox in Preventing Infection and Progression

**DOI:** 10.3390/jcm9041126

**Published:** 2020-04-15

**Authors:** Florence Carrouel, Maria Pia Conte, Julian Fisher, Lucio Souza Gonçalves, Claude Dussart, Juan Carlos Llodra, Denis Bourgeois

**Affiliations:** 1 University Lyon 1, Laboratory “Health Systemic Process”, EA4129, 69008 Lyon, France; denis.bourgeois@univ-lyon1.fr; 2 Department of Public Health and Infectious Diseases, Sapienza University of Rome, 00185 Rome, Italy; mariapia.conte@uniroma1.it; 3 THEnet, Training for Health Equity Network, New York, NY 10023, USA; julian.fisher@thenetcommunity.org; 4 Faculty of Dentistry, Estacio de Sá University, Rio de Janeiro 20261-063, Brazil; lucio.souza@estacio.br; 5 Lyon Public Hospices, Central Pharmacy, EA4129, 69002 Lyon, France; claude.dussart@univ-lyon1.fr; 6 Department of Preventive and Community Dentistry, University of Granada, 18010 Granada, Spain; jllodra@hotmail.com

**Keywords:** COVID-19, 2019-nCoV, SARS-CoV-2, oral cavity, mouthrinse, β-cyclodextrins, Citrox, viral load, microbiome

## Abstract

Considered to be a major portal of entry for infectious agents, the oral cavity is directly associated with the evolutionary process of SARS-CoV-2 in its inhalation of ambient particles in the air and in expectorations. Some new generations of mouth rinses currently on the market have ingredients that could contribute to lower the SARS-CoV-2 viral load, and thus facilitate the fight against oral transmission. If chlorhexidine, a usual component of mouth rinse, is not efficient to kill SARS-CoV-2, the use of a mouth rinses and/or with local nasal applications that contain β-cyclodextrins combined with flavonoids agents, such as Citrox, could provide valuable adjunctive treatment to reduce the viral load of saliva and nasopharyngeal microbiota, including potential SARS-CoV-2 carriage. We urge national agencies and authorities to start clinical trials to evaluate the preventive effects of βCD-Citrox therapeutic oral biofilm rinses in reducing the viral load of the infection and possibly disease progression.

## 1. Introduction

The epidemic of infection COVID-19 (or 2019-CoV) by an emerging coronavirus SARS-CoV-2 in December 2019 has generated severe threats to international health security, global health, and the economy [1]. Given the current lack of effective treatment there is a need to explore alternative methods to contain the propagation of the infection, focusing in particular on its mode of transmission. The person-to-person modes of transmission of SARS-CoV-2 are direct transmissions, such as sneezing, coughing, transmission through inhalation of small droplets, and transmission by contact such as contact with nasal, oral, and ocular mucous membranes [2]. SARS-CoV-2 may also be transmitted directly or indirectly by the saliva, and the fetal–oral routes can be a possible route of person-to-person transmission as well [3,4]. Moreover, high viral loads have been found in the oropharynx of infected patients, as well as in the asymptomatic subjects [5]. This could suggest that the potential of SARS-CoV-2 transmission is wider than originally thought. The oral cavity is therefore directly associated with the evolutionary process of SARS-CoV-2 in its inhalation of ambient particles in the air and in expectorations.

Considered to be a major portal of entry for infectious agents [6], the oral cavity is colonized by a large number and variety of micro-organisms, including bacteria, fungi, and viruses termed microbiota [7]. These microbial communities and their inevitable multiple synergistic and antagonistic interactions are reflected in an individual’s oral and general health. The oral cavity and nasopharyngeal regions can be considered as the anatomical transition between external and internal environments. The standard oral cavity temperature is on average 37 °C with no notable variations, which gives the microorganisms a secure environment to survive [8]. Saliva is also pH stable at 6.5–7, which is the favorable pH to oral microbiota (bacteria species and virus such as coronavirus) [9,10,11]. Variations in saliva composition are often associated with microbiota dysbiosis and oral diseases [12]. Moreover, salivary composition influences oropharyngeal colonization characteristics and bacterial profile [13]. Oral microbiota comprises of commensal bacterial populations that sustain mutual benefits with the host and keep potentially pathogenic bacteria in balance through a number of negative feedback mechanisms. In the oral biofilm, these bacteria combine to make a barrier which resists to antibiotics, disinfectants, mechanical removal, and other stresses [14]. In addition, bacteria within biofilms have 1000 times more resistance to antibacterial treatments compared to planktonic microorganisms [15]. In the absence of proper oral hygiene, the percentage of potentially health damaging bacteria in biofilm are seen to increase, contributing to the development of chronic infection [16]. Moreover, in biofilms, bacteria can escape the immune system producing so-called superantigens [17]. In addition to host–microbe interactions, the interfaces of periodontal pathogens with other non-host pathogens, such as herpesviruses like Epstein–Barr virus and cytomegalovirus, can contribute to the pathogenesis of the periodontal disease [18], or can affect the outcome of viral infection and dissemination [19]. These risks are often significantly underestimated. In the future, oral care measures that are effective in reducing infection should be given higher priority.

## 2. Mouth Rinses for Infection Containment

There is currently a large variety of over-the-counter mouth rinses available, which contain a wide range of active ingredients with for each having specific indication [20]. Cosmetic mouth rinses can temporary control halitosis and taste pleasant, but have no biological or chemical application that goes beyond their immediate benefit [21]. On the other hand, therapeutic mouth rinses include active ingredients that are designed to modify the oral microbiota for conditions such as gingivitis, dental caries, plaque, and bad breath. A new generation of therapeutic oral biofilm rinses developed to control virulent bacteria have added metals, metal oxides, and other nanoparticles, which appear to be promising alternatives due to their distinct physio–chemical properties [20].

### 2.1. Chlorhexidine

Effective ingredients that may be applied in therapeutic mouth rinses includes: chlorhexidine (CHX), fluoride, cetylpyridinium chloride, essential oils, and peroxide [22]. Conceived for short-term usage, CHX 2% is a cationic bisbiguanide widely used in general medical practice as a broad-spectrum antiseptic. CHX increases the permeability of the bacterial cell wall, resulting in bacterial lysis [23]. Its activity includes gram-positive and gram-negative bacteria (gram-positive bacteria being more susceptible), aerobes, facultative anaerobes, fungi, and selected viruses [24]. While chlorhexidine significantly reduces the risk of ventilator-associated pneumonia, no differences were found in terms of mortality, mechanical ventilation, or length of stay in the intensive care unit [25]. However, and of major importance for the care of ventilator-associated COVID-19 patients, several drawbacks have been cited i.e., a reduced susceptibility to CHX of number of ventilator-associated pneumonia pathogens and an increased risk of death in the less severe patients [23]. 

In oral health, it is commonly accepted that a preoperative antimicrobial mouth rinse decreases the number of oral pathogens. One of the more common CHX indications are gingivitis, periodontitis, post-surgery periodontal disease, and implantology [26]. While CHX at lower concentrations is bacteriostatic, at higher concentrations it also has a bactericidal effect. CHX has been reported to penetrate the oral biofilms as well, affecting their growth or directly having a bactericidal impact [27]. CHX nanoparticles have the potential to inhibit the development of a multi-species oral biofilm made up of *Porphyromonas gingivalis*, *Streptococcus sobrinus*, and *Fusobacterium nucleatum* [28].

### 2.2. Flavonoids

Flavonoids constitute an important category of natural products and include various subgroups, such as flavones, chalcones, isoflavones, and flavonols [29]. Bioflavonoids are phenolic hydroxylated structures that have been synthesized from plants and have been shown to be active against fungi, bacteria, and viruses [30,31]. Flavonoids are a group of phytochemicals with a wide range of biological activities, mainly due to their antioxidant properties and their ability to regulate several cell receptors or enzymes [29]. Flavonoids have also been identified as having antiviral activity, antibacterial and anti-inflammatory effect, anti-allergic and antiangiogenic effects, and cytostatic, analgesic, apoptotic, hepatoprotective, antiestrogenic, and estrogenic properties [30,32,33,34]. Shimizu et al., discovered that flavonoids of Pterogyne nitens were able to inhibit the entry of the hepatitis C virus [35]. Jo et al., also suggested that the anticoronaviral activity of certain flavonoids (Rhoifolin, herbacetin, and pectolinarin) was attributable to the inhibitory effect of the 3C protease type [36]. Some others flavonoids (Isobavachalcone, herbacetin, helichrysetin, quercetin, and 3-β-d-glucoside) were able to inhibit the enzyme activity of MERS-CoV/3CLpro [36]. Ryu et al., in addition, reported that the biflavonoids of Torreya nucifera were also inhibitors of MERS-CoV/3CLpro [37].

To achieve a stronger impact, CHX mouth rinses were combined with plant products, as previous research has reported the efficient and effective utilization of natural antimicrobials inhibiting oral microbiota [38]. Several types of oral rinses are currently available, with additional ingredients containing mainly medical plants, alcohol, and natural substances [39]. More specifically, oropharyngeal microbiota has been identified as being involved in ventilator-associated pneumonia, and the utilization of therapeutic formulations using essential oils can decrease the incidence of this significant infection [40]. In vitro investigations were conducted examining the antimicrobial activities of five various mouth rinses using 14 test strains comprising *Candida* species and bacteria, cultivated both in plankton and as biofilms.

In four of these mouth rinses formulations, Citrox, a combination of natural bioflavonoids, was present. Three of these Citrox mouth rinses were complemented with hyaluronic acid, chlorhexidine or phenoxetol [41]. Citrox is an antimicrobial with ingredients based on natural soluble bioflavonoids extracted of citrus fruits. Citrox bioflavonoid preparations have a broad spectrum of antimicrobial activity on oral microorganisms and as such can be used within therapeutic formulations for the oral microbiota control [40].

### 2.3. Cyclodextrins

Cyclodextrins (CDs) are natural derivatives of glucose, with a rigid cyclic structure, composed of α(1-4)–linked gluco-pyranoside units [42]. The most usual CDs, called, α, β, and γ, contain 6-, 7-, and 8-glucopyranoside units, respectively. CDs are cyclic oligosaccharides used for improving bioavailability of medicinal products and water-solubility [43]. Also, CDs can be employed to prevent or reduce ocular and gastrointestinal irritation, decrease or eliminate disagreeable tastes or smells, prevent interactions between drugs or drug additives in a formulation [44]. They have been used in a multitude of commercial sectors, such as deodorants, drug delivery, food, and cosmetics. On the European market, examples of use of CDs in drugs are γ-CD in a minoxidil solution, β-CD in cetirizine tablets and cisapride suppositories and examples of use of derivatives of β-CD derivatives are HP-β-CD in itraconazole antifungal, in intravenous and oral solutions, SBE-β-CD in intravenous voriconazole antimycotic, and RM-β-CD in a nasal spray for hormone replacement therapy with 17β-estradiol [45].

Cyclodextrins have many advantages. They are more biocompatible than most oxides contained in oral products (i.e., silver and gold), simpler to use, they do not generate a resistance reaction, and they are not toxic [46]. Cyclodextrins have no harmful effects and are considered “generally regarded as safe” for humans [46]. The fields of pharmaceutical application of CDs are notable due to their low immunogenicity, low toxicity, cost-effectiveness, and accessibility [47]. These applications include: increasing drug stability and solubility, improving drug absorption, masking undesirable tastes and odors, controlling drug release, eliminating local and systemic toxicity, and improving drug permeability via biological barriers [48,49]. 

Their strucutre of cyclodextrins can be modified and used for containment of infections or as virucidal agents [50]. For example, it has been established that methylated beta-cyclodextrin can reduce influenza A virus and coronavirus infectivity by sequestering cholesterol from viral particles or depleting it from the host cell membranes [51]. Hydroxypropyl-β-cyclodextrin was used as a vaccine adjuvant providing protection against the H1N1 influenza virus in the cynomolgus monkey model [52,53]. One point to emphasize is that for natural CDs, the intramolecular hydrogen bond with the CD molecule decreases their hydrogen bond formation with the surrounding water molecules [54]. This could lead to a potential negative antiviral action against the coronavirus.

Recently, Jones et al. further developed the concept of cyclodextrins modified with mercaptoundecane sulfonic acids to provide the key nontoxic virucidal action and to mimic heparan sulfates. Very promising studies have indicated that the modified sugar molecules attract viruses before irreversibly inactivating them [55]. By disrupting the outer shell of a virus, cyclodextrins, modified with mercaptoundecane sulfonic acids can destroy infectious particles by simple contact, rather than simply blocking viral growth. This mechanism seems to be the same regardless of the virus concerned. These modified cyclodextrins are biocompatible, broad-spectrum, and virucidal at in vitro micromolar concentrations against many viruses, including respiratory syncytial virus (RSV), herpes simplex virus (HSV), Zika virus, and dengue virus. They are effective ex vivo against both laboratory and clinical strains of RSV and HSV-2 in respiratory and vaginal tissue culture models, respectively [55].

Amphiphilic β-cyclodextrin nanoparticles (C42H70O35) with seven glucose units has the mid-size cavity of the parent cyclodextrins, has been added to the composition of commercial mouth rinses to prevent flocking out of hyaluronic acid in the combination of CHX-Polylysine and hyaluronic-acid. Two commercial mouth rinses including β-CD are today available.

Amphiphilic CDs, useful for solubilizing, stabilizing, or releasing intermediate-sized molecules, have been produced by synthesis to solve multiple difficulties of parent cyclodextrins that restrict their pharmaceutical uses [56]. The main justifications for the synthesis of amphiphilic CDs were to: improve the interactions of cyclodextrins with biological membranes, increase the interaction of CDs with hydrophobic drugs, and enhance self-assembly capability in aqueous solutions [57].

## 3. Components of Mouth rinses and CoVID-19-Specific Treatment

In the absence of vaccines or medicines that will unfortunately arrive too late for many patients, it will be crucial to explore how existing treatments might be used as coronavirus specific interventions. Some mouth rinses currently on the market have ingredients that could contribute to the reduction of the SARS-CoV-2 viral load and thus facilitate the fight against oral transmission. We urge consideration be given to the following statements. While we recognize these statements do not have coronavirus specific evidence, they nonetheless can provide valuable information and insight to health workers and can support system-wide efforts to rapidly identify effective treatment protocols.

### 3.1. Mouth Rinses with Chlorhexidine for CoVID-19-Specific Treatment

In accordance with the Guidelines for the Diagnosis and Treatment of New Coronavirus Pneumonia (5th edition) of the National Health Commission of the Republic of China, CHX as mouth rinse may not be efficient to kill SARS-CoV-2 [4].

### 3.2. Mouth Rinses with Citrox for CoVID-19-Specific Treatment

Flavonoids as coronaviral chymotrypsin-like protease inhibitors have an essential function for coronaviral replication and also have additional functions for inhibition of host innate immune responses and should be useful in fighting COVID-19 [1]. Moreover, as SARS-CoV-2 is vulnerable to oxidation, it is recommended to use a mouth rinse containing oxidizing agents such as Citrox to reduce the salivary load viral of oral microbiota, including potential SARS-CoV-2 carriage.

### 3.3. Mouth Rinses with Amphiphilic β-Cyclodextrin for CoVID-19-Specific Treatment

As for the justification for the use of Citrox delivery, and the fact that SARS-CoV-2 is vulnerable to oxidation, mouth rinses containing oxidative agents such as amphiphilic CDs, appear indicative for the purpose of reducing the salivary load of oral microbes. The modified sugar molecules attract viruses before irreversibly inactivating them. By disrupting the outer shell of a virus, they can destroy infectious particles by simple contact, rather than just blocking viral growth [20]. This property of β-CDs can potentially be exploited for the reduction of viral load in the oral cavity with the use of disinfectant solutions. In addition, the use of therapeutic oral biofilm rinses and/or nasal applications might be considered in preventing viral transmission via the oropharyngeal route.

### 3.4. Mouth Rinses with Cyclodextrins Combined with Citrox for CoVID-19-Specific Treatment

The use of a mouth rinses and/or nasal applications that contain cyclodextrins combined with Citrox could provide a valuable adjunct treatment. Both are locally administered delivery systems that could lower the SARS-CoV-2 viral load and reduce the nasopharyngeal microbiota, which tends to coat the surface aerosol particles and droplets during coughing or sneezing.

Such products are available in Europe as oral health products and have been well tested in clinical profiles. We urge national agencies and authorities to start clinical trials to evaluate the preventive effects of βCD-Citrox therapeutic oral biofilm rinses in reducing the viral load of the infection and possibly disease progression. 

We ask that consideration is given to CDs combined with Citrox in the form of therapeutic oral biofilm rinses and/or nasal applications, which can augment other approaches and treatment modalities.
